# MR-proADM as prognostic factor of outcome in COVID-19 patients

**DOI:** 10.1038/s41598-021-84478-1

**Published:** 2021-03-04

**Authors:** Emanuela Sozio, Carlo Tascini, Martina Fabris, Federica D’Aurizio, Chiara De Carlo, Elena Graziano, Flavio Bassi, Francesco Sbrana, Andrea Ripoli, Alberto Pagotto, Alessandro Giacinta, Valentina Gerussi, Daniela Visentini, Paola De Stefanis, Maria Merelli, Kordo Saeed, Francesco Curcio

**Affiliations:** 1 U.O. Malattie Infettive, Dipartimento Di Medicina Dell’Università Di Udine, Università Di Udine E Azienda Sanitaria Universitaria Integrata Di Udine, Via Pozzuolo, 330, 33100 Udine, Italy; 2 Istituto Di Patologia Clinica, Azienda Sanitaria Universitaria Integrata Di Udine (ASUID), Udine, Italy; 3 SOC Anestesia E Rianimazione 2, Università Di Udine E Azienda Sanitaria Universitaria Integrata Di Udine, Udine, Italy; 4 U.O. Lipoapheresis and Center for Inherited Dyslipidemias, Fondazione Toscana Gabriele Monasterio, Pisa, Italy; 5 Deep Health Unit, Fondazione Toscana “Gabriele Monasterio”, Pisa, Italy; 6 Microbiology Innovation and Research Unit (MIRU), Department of Microbiology, University Hospitals Southampton NHS Foundation Trust, and University of Southampton School of Medicine, Southampton, UK

**Keywords:** Infectious diseases, Pathogenesis, SARS-CoV-2

## Abstract

Mid Regional pro-ADM (MR-proADM) is a promising novel biomarker in the evaluation of deteriorating patients and an emergent prognosis factor in patients with sepsis, septic shock and organ failure. It can be induced by bacteria, fungi or viruses. We hypothesized that the assessment of MR-proADM, with or without other inflammatory cytokines, as part of a clinical assessment of COVID-19 patients at hospital admission, may assist in identifying those likely to develop severe disease. A pragmatic retrospective analysis was performed on a complete data set from 111 patients admitted to Udine University Hospital, in northern Italy, from 25th March to 15th May 2020, affected by SARS-CoV-2 pneumonia. Clinical scoring systems (SOFA score, WHO disease severity class, SIMEU clinical phenotype), cytokines (IL-6, IL-1b, IL-8, TNF-α), and MR-proADM were measured. Demographic, clinical and outcome data were collected for analysis. At multivariate analysis, high MR-proADM levels were significantly associated with negative outcome (death or orotracheal intubation, IOT), with an odds ratio of 4.284 [1.893–11.413], together with increased neutrophil count (OR = 1.029 [1.011–1.049]) and WHO disease severity class (OR = 7.632 [5.871–19.496]). AUROC analysis showed a good discriminative performance of MR-proADM (AUROC: 0.849 [95% Cl 0.771–0.730]; *p* < 0.0001). The optimal value of MR-proADM to discriminate combined event of death or IOT is 0.895 nmol/l, with a sensitivity of 0.857 [95% Cl 0.728–0.987] and a specificity of 0.687 [95% Cl 0.587–0.787]. This study shows an association between MR-proADM levels and the severity of COVID-19. The assessment of MR-proADM combined with clinical scoring systems could be of great value in triaging, evaluating possible escalation of therapies, and admission avoidance or inclusion into trials. Larger prospective and controlled studies are needed to confirm these findings.

## Introduction

The pathological mechanisms of organ damage in Coronavirus disease 2019 (COVID-19) patients remain poorly understood. The leading cause of mortality in patients with COVID-19 is hypoxic respiratory failure from acute respiratory distress syndrome (ARDS)^1^. The multiple organ failure described in COVID-19 suggests a multi-pathway involvement.

Evidence suggests that pulmonary endothelial cells (ECs) contribute to the initiation and propagation of ARDS by altering vascular barrier integrity, promoting a pro-coagulative state, inducing vascular inflammation (endotheliitis), tissue edema and mediating inflammatory cell infiltration^2^. Severe Acute Respiratory Syndrome Coronavirus two (SARS-CoV-2) virus binds Angiotensin Converting Enzyme 2 (ACE2) receptor and induces a down-regulation of ACE2 that results in activation of kallikrein–bradykinin pathway, increasing vascular permeability^3^.

Cytokine storm has been postulated in COVID-19, at least in the last phase during ARDS^4,5^ amplifying the destructive process by leading to further endothelial cells dysfunction and vasodilation of the pulmonary capillary bed^6,7^. The experiences of Sinha et al. and of Kox et al. showed elevated IL-6 levels in severe COVID-19, as observed in other critically ill patients; at the same time, IL-6 levels in COVID-19 were lower than in patients with bacterial sepsis^4,5^.

The alteration of vascular barrier integrity, the pro-coagulative state, endotheliiyis and the cytokine storm might contribute to the progression towards ARDS and ultimately to multi-organ failure and death^8^.

The role of biomarkers such as procalcitonin (PCT) and C-reactive protein (CRP) is already well established in infections; the PCT and CRP dosing are routinely used to confirm the presence of infection and in the follow-up during medical treatment. Recently, Adrenomedullin (ADM) or, more precisely, its stable protein surrogate Mid Regional pro-Adrenomedullin (MR-proADM), gained interest as a major player in deteriorating patients. MR-proADM plays a role in vascular permeability, inflammatory mediation, endothelial barrier regulation and stabilization of the microcirculation, all of which contribute to the development of organ dysfunction and failure in sepsis and septic shock. Therefore, the increase of MR-proADM is seen as an indicator of organ dysfunction and, unlike most other biomarkers, it is essential in maintaining endothelial stability. For this reason, it may be a relevant biomarker to understand the ECs.

Saeed et al. have studied MR-proADM in patients presenting to the emergency department with a suspected infection and they proposed two major clinical uses for it: 1) An early escalation of antibiotic and resuscitation treatment in patients with MR-proADM values > 1,5 nmol/L, as such high MR-proADM concentrations identify severe illness and a potential for further progression of the disease; 2) A potential reduction of the number of hospitalizations and re-admissions for patients with MR-proADM values < 0,9 nmol/L, that is the absence of endothelial damage^9^. MR-proADM identifies disease severity and treatment response more accurately than established biomarkers and scores indeed [ 10].

MR-proADM may also be of interest within COVID-19 induced endotheliitis^11^, but only two studies have described the potential role of this biomarker in COVID-19 patients so far. The first study evaluated only 20 patients with COVID-19, measuring adrenomedullin RNA expression and the authors found a correlation between RNA expression and severity of the disease^12^. In the second study, Montruccio et al.found higher levels of MR-proADM in COVID-19 patients admitted to Intensive Care Unit (ICU) and hence they suggested that MR-proADM can be used as a predictor factor of disease progression and mortality, with a cut-off value > 1,8 nmol/L^13^.

At Udine University Hospital, a number of biomarkers including MR-proADM and other cytokines have been implemented during the first wave of COVID-19 outbreak.

We hypothesized that the assessment of MR-proADM, with or without other inflammatory cytokines, as part of a clinical assessment of COVID-19 patients at hospital admission, may assist in identifying those likely to develop severe disease, facilitating triaging and decision-making about possible treatment escalations.

## Materials and methods

### Patient population

A pragmatic retrospective analysis of 111 COVID-19 patients admitted to Udine University Hospital, in northeast Italy, from 25th March to 15th May 2020, was conducted. All the included cases had the diagnosis of SARS-CoV-2 pneumonia and for all of them was possible to make a complete review of the data set.

All patients enrolled had positive PCR test for SARS-CoV-2 and features of respiratory infection, evaluated with radiologic chest imaging (chest CT, chest XR and/or Point-of-Care Ultrasound) and other clinical signs.

SARS-CoV-2 infection status was evaluated by PCR on nasopharyngeal swabs. The mean interval between symptom presentation and swab sample collection was found to be 6 days [CI_95_ 3–9].

The full spectrum of COVID-19 ranged from mild, self-limiting respiratory tract illness, to severe progressive pneumonia, multi-organ failure, and death. The clinical severity of patients suffering from COVID-19 was evaluated using the classification reported on World Health Organization guidance^14^ and the Italian Society of Emergency and Urgency Medicine (SIMEU) clinical phenotypes^15^.

For all the patients enrolled in this study the following parameters were recorded: age, gender, Charlson comorbidity index, length of hospital stays, sequential organ failure assessment score value (SOFA score) at admission, clinical severity as already described, and PaO_2_/FiO_2_ at admission. Furthermore, during hospitalization, the following markers were measured in all the included patients: white blood cells count, encompassing neutrophils, lymphocytes and the ratio CD4/CD8, platelets count, creatinine, bilirubin, CRP, PCT, D-dimer, lactate dehydrogenase (LDH), Creatine Kinase (CK), B-type natriuretic peptide, high-sensitive cardiac troponin, IL-6, IL-1b, IL-8, TNF-α, and MR-proADM.

A negative outcome was defined by a combination of death and orotracheal intubation (IOT) during hospitalization.

All research was performed in accordance with the relevant guidelines and regulations. This study was approved by the Friuli Venezia Giulia ethics committee and the informed and written consent was obtained from the participants.

### Laboratory methods

Real-time reverse transcriptase-PCR assays for the detection of SARS-CoV-2 on upper respiratory specimens collected from swabs were conducted at the Virology and Microbiology Laboratories of Udine, according to WHO guidance^16^.

MR-proADM plasma concentrations were measured in an automated Kryptor analyzer, using TRACE technology (Kryptor; BRAHMS, Hennigsdorf, Germany). The lower detection limit was 0.05 nmol/L, while the limit Of Quantitation (LOQ) was 0.23 nmol/L. The cut-off for physiological concentration was pointed at 0.56 nmol/L. Cytokines were measured by microfluidic ultrasensitive ELISA using the Protein simple plex technology on ELLA instrument (R&D systems, Biotechne, USA). The ranges of quantitation are as follows: IL-1β (0.16–1530 pg/ml), IL-6 (0.28–2652 pg/ml), IL-8 (0.19–1804 pg/ml), TNF-α (0.30–1160 pg/ml). All the other laboratory biomarkers were evaluated using routine certified diagnostic methods.

### Statistical analysis

Baseline patient characteristics were summarized using standard descriptive statistics, with number and percentages for binary and categorical outcomes and appropriate measures for continuous outcomes (e.g. mean ± standard deviation or median and interquartile range, depending on their distribution). Accordingly, comparisons between groups were performed with independent sample t-test, Mann–Whitney U test or chi-squared test with continuity correction. The relation between outcome and acquired variables was investigated with a logistic regression model; the covariates with *p* < 0.10 at the univariate logistic regression were considered for a multivariate stepwise model. Considering the small sample size and the strong correlation structure observed in the data, the multivariate regression was computed on a subset of the considered covariate. This further process of covariate selection was based on the statistically equivalent signature algorithm^17^.

Receiver-operator characteristic (ROC) analysis was used to determine the diagnostic performance of MR-proADM; jack-knife estimates of sensitivity and specificity were reported at the optimal cutoff point, chosen with Youden's rule.

All analyses were performed with the R statistical software^18^. A *p* value of less than 0.05 was considered to be statistically significant.

### Study endpoint

Severe disease or negative outcome assessed by the composite end point: the use intubation mechanical ventilation and/or death.

## Results

During the study period (25th March–15th May 2020), 111 patients with COVID-19 were enrolled. Patient characteristics and demographic are summarized in Table 1. The comparison between favorable outcome (83 patients) and negative outcome (28 patients) found that factors associated with the second group were (see Table 1): gender, higher WHO disease severity class, SIMEU disease severity phenotype, higher SOFA score, lower PaO_2_/FiO_2_ ratio, lower lymphocytes count, higher level of MR-proADM, IL-6, IL-1b, IL-8, TNFα, white blood cells, neutrophils, LDH, CK and C reactive protein. The length of stay was higher in the group with negative outcome.

**Table 1:** Overall study population and comparison between the group with combined event of Death or IOT and the group without combined event.

	Overall (n = 111)	Not death and not IOT (n = 83)	Death or IOT (n = 28)	*p*
Males	66 (59.5%)	44 (53.0%)	22 (78.6%)	0.0308
Age (years)	62.29 ± 13.63	61.73 ± 14.28	63.93 ± 11.6	0.4191
WHO disease severity	2.24 ± 1.03	1.86 ± 0.72	3.39 ± 0.96	< 0.0001
SIMEU disease severity	2 [2–3]	2 [2–3]	5 [4–5]	< 0.0001
PaO_2_/FiO_2_ ratio	292.5 ± 109.4	330.9 ± 85.6	183.2 ± 96.2	< 0.0001
SOFA score	2 [1–3]	2 [1–2]	3 [3–4]	< 0.0001
Charlson Comorbidity Index	2 [1–4]	2 [1–4]	2.5 [1.75–4]	0.4477
Length of hospital stay (days)	9 [6–22]	7 [4.5–10]	30 [23–47]	< 0.0001
MR-proADM (nMol/L)	0.82 [0.64–1.08]	0.73 [0.56–0.94]	1.38 [0.94–1.73]	< 0.0001
IL-6 (pg/mL)	30.0 [7.7–82.3]	21.9 [4.2–51.0]	175.7 [48.0–1070.0]	< 0.0001
IL-1b (pg/ml)	0.33 [0.18–0.48]	0.28 [0.15–0.42]	0.48 [0.26–0.88]	0.0069
IL-8 (pg/mL)	32.0 [21.0–43.1]	27.7 [18.3–38.4]	37.5 [30.0–62.6]	0.0018
TNFα (pg/mL)	17.0 [13.3–21.8]	16.0 [12.8–19.1]	23.7 [17.6–31.4]	0.0001
C reactive protein (mg/dl)	71.0 [16.9–117.0]	48.0 [10.3–99.5]	108.5 [72.3–200.8]	0.0002
Procalcitonine (mg/dL)	0.07 [0.02–0.29]	0.04 [0.02–0.14]	0.31 [0.18–0.47]	< 0.0001
White blood cell (/mmc)	6020 [4745–7925]	5760 [4330–7465]	7030 [5892–12725]	0.001
Neutrophils (/mmc)	4420 [3130–6680]	3900 [2720–5945]	6175 [4898–11510]	< 0.0001
Lymphocytes (/mmc)	914 ± 435	1007 ± 442	639 ± 268	< 0.0001
CD4/CD8	2.1 [1.4–2.9]	2.0 [1.3–2.9]	2.2 [1.8–4.2]	0.1016
D–dimer (FEUng/ml)	751 [403–1200]	690 [343–1045]	1157 [759.5–1959]	0.0008
LDH (U/L)	517 [375–735]	452 [350–646]	758 [637–951]	< 0.0001
CK (U/L)	92 [55–178]	78 [53–127]	185 [106–333]	0.0002
B-type natriuretic peptide (pg/ml)	28 [12–66]	25 [11–63]	34 [20–92]	0.2634
High-sensitive cardiac troponin (ng/L)	0.02 [0.00–0.02]	0.02 [0.00–0.02]	0.02 [0.00–0.02]	0.3407
Bilirubin (mg/dl)	0.55 [0.40–0.78]	0.51 [0.38–0.78]	0.60 [0.50–0.78]	0.1638
Platelets (/mmc)	224,189 ± 98,333	220,398 ± 85,808	235,429 ± 129,834	0.571
Creatinine (mg/dl)	1.00 ± 0.48	0.98 ± 0.51	1.08 ± 0.37	0.2636

Factors influencing negative outcome considered as combined events of death or IOT were studied with univariate and multivariate analysis and these are summarized in Table 2.

**Table 2:** Factors influencing death or IOT (univariate & multivariate analysis).

**Variables**	**Univariate analysis**		**Multivariate analysis**
	**OR [95% CI]**	***p***	**OR [95% CI]**
Males	3.25 [1.2565–9.5691]	0.0209	–
Age (years)	1.0122 [0.9806–1.0465]	0.4607	–
WHO disease severity	8.55 [4.1473–21.841]	< 0.0001	7.632 [5.871–19.496]
SIMEU disease severity	4.9577 [3.0004–9.3041]	< 0.0001	–
PaO_2_/FiO_2_ ratio	0.9816 [0.9731–0.9883]	< 0.0001	–
SOFA score	4.1475 [2.4822–7.9195]	< 0.0001	–
Charlson Comorbidity Index	1.0953 [0.9548–1.2742]	0.1949	–
Length of hospital stay (days)	1.1121 [1.0697–1.168]	< 0.0001	–
MR-proADM (nMol/L)	4.329 [1.9178–12.4701]	0.0024	4.284 [1.893–11.413]
IL-6 (pg/mL)	1.0081 [1.0038–1.0146]	0.0025	–
IL-1b (pg/ml)	0.9702 [0.5885–1.2803]	0.8516	–
IL-8 (pg/mL)	1.0119 [0.9995–1.0289]	0.0979	–
TNFα (pg/mL)	1.1302 [1.0637–1.2223]	0.0005	–
C reactive protein (mg/dl)	1.0107 [1.0053–1.0169]	0.0003	–
Procalcitonine (mg/dL)	1.0236 [0.7832–1.2445]	0.7954	–
White blood cell (/mmc)	1.0187 [1.0081–1.0316]	0.0015	–
Neutrophils (/mmc)	1.0243 [1.0119–1.0394]	0.0004	1.029 [1.011–1.049]
Lymphocytes (/mmc)	0.9967 [0.9947–0.9983]	0.0002	–
CD4/CD8	1.3727 [1.0469–1.8571]	0.028	–
D –dimer (FEUng/ml)	1.0112 [0.9981–1.0283]	0.1136	–
LDH (U/L)	1.4282 [1.2105–1.7343]	0.0001	–
CK (U/L)	1.3014 [1.0344–1.6797]	0.0296	–
B-type natriuretic peptide (pg/ml)	1.3294 [1.0285–1.9537]	0.0838	–
High-sensitive cardiac troponin (ng/ml)	1.0306 [1.005–1.0675]	0.0431	–
Bilirubin (mg/dl)	2.4935 [0.8597–7.5184]	0.0902	–
Platelets (/mmc)	1.0015 [0.9971–1.0057]	0.4843	–
Creatinine (mg/dl)	1.477 [0.6115–3.9612]	0.359	–

The variables selected for multivariate analysis were: gender, WHO disease severity, MR-proADM, PaO2/FiO2 ratio, IL-6, CD4/CD8, Neutrophils count.

The univariate analysis confirmed that factors associated with negative outcome were (see Table 2): to be male (OR 3.25 [1.2565–9.5691]; *p* = 0.0209), have an higher WHO disease severity class (OR 8.55 [4.1473–21.841]; *p* < 0.0001) and SIMEU disease severity phenotype (OR 4.9577 [3.0004–9.3041]; *p* < 0.0001), higher SOFA score (OR 4.1475 [2.4822–7.9195]; *p* < 0.0001), lower PaO_2_/FiO_2_ ratio at admission (OR 0.9816 [0.9731–0.9883]; *p* < 0.0001), longer the length of hospital stay (OR 1.1121 [1.0697–1.168]; *p* < 0.0001), higher level of MR-proADM (OR 4.329 [1.9178–12.4701]; *p* = 0.0024), IL-6 (OR 1.0081 [1.0038–1.0146]; *p* = 0.0025), TNFalfa (OR 1.1302 [1.0637–1.2223]; *p* = 0.0005), C-reactive protein (OR 1.0107 [1.0053–1.0169]; *p* = 0.0003), white blood cells count (OR 1.0187 [1.0081–1.0316]; *p* = 0.0015), neutrophils count (OR 1.0243 [1.0119–1.0394]; *p* = 0.0004), LDH (OR 1.4282 [1.2105–1.7343]; *p* = 0.0001), CK (OR 1.3014 [1.0344–1.6797]; *p* = 0.0296), bilirubin (OR 2.4935 [0.8597–7.5184]; *p* = 0.0902), lower levels of lymphocytes (OR 0.9967 [0.9947–0.9983]; *p* = 0.0002).

The multivariate analysis was able to found three factors associated with negative outcome (see Table 2): higher WHO disease severity class (OR = 7.632 [5.871–19.496]; *p* < 0.0001), MR-proADM (OR = 4.284 [1.893–11.413]; *p* = 0.0006) and neutrophils count (OR = 1.029 [1.011–1.049]; *p* = 0.0018).

**Figure 1:**
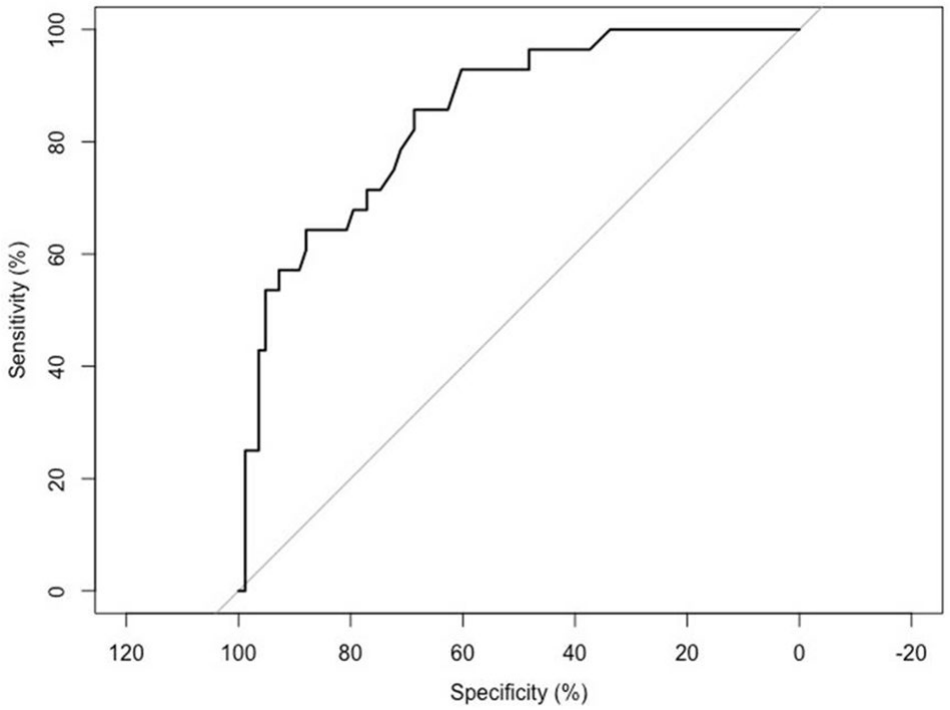
AUROC analysis: MR-proADM on combined event of death or orotracheal intubation (IOT). AUROC: 0.849 [0.771–0.730], *p* < 0.0001. Optimal cut-off value of MR-proADM: 0.895; a sensitivity of 0.857 [0.728–0.987] and a specificity of 0.687 [0.587–0.787] correspond to this cutoff value.

AUROC analysis showed a good discriminative performance of MR-proADM with respect to the combined event of death or IOT (AUROC: 0.849 [95% Cl 0.771–0.730]; *p* < 0.0001, see Fig. 1). The optimal value of MR-proADM, to discriminate combined event of death or IOT from the group of patients which had a favorable outcome (not dead and not IOT) is 0.895 nmol/l; a sensitivity of 0.857 [95% Cl 0.728–0.987] and a specificity of 0.687 [95% Cl 0.587–0.787] correspond to this cut-off value of 0.895 nmol/l.

## Discussion

We aimed to understand the pathophysiologic behind endothelial damage that might drive a more severe clinical deterioration by evaluating MR-ProADM and other cytokine levels at the point of diagnosis. These protein molecules may be used as a guide to differentiate disease severity and to manage the infection. Our findings suggest that among all biomarkers and cytokines we looked at, MR-proADM was significantly higher in patients hospitalized with COVID-19 and with negative outcome.

MR-proADM could be incorporated as a biomarker in assessing COVID-19 pneumonia severity. MR-proADM identifies disease severity and treatment response more accurately than established biomarkers (e.g. PCT and CRP) and clinical scores in septic patients^10^. Clinical scores such as the SOFA score were developed to assess the severity of critical patients; specifically, the SOFA score is part of the new definition of sepsis too^19^. This score is not able to capture individual organ system dysfunction and it is not used in daily routine for COVID-19. A quantitative biomarker, if effective, might be more useful than scores in predicting severity of patients. The use of a relatively novel biological markers like MR-proADM, which is significantly increased during the initial stages of sepsis development, may therefore help in determining or predicting disease severity, guide early diagnostic interventions and facilitate more intensive treatment in severe cases before the establishment of further organ dysfunction, regardless of the causative agent (bacteria, fungi or viruses)^20–22^.

Moreover, oxygen requirement/saturation as parameters have been used as triaging tool for COVID-19 patients during hospital admission. However, when supplemented oxygen has been instituted before an intensivists’ evaluation, measuring the PaO_2_/FiO_2_ ratio can become very challenging (actually it is known that, if patients are not intubated, is almost impossible to know the exact fractional inspired oxygen concentration (FiO_2_)). For instance, with a nasal cannula set at 2 L/minute, FiO_2_ may range anywhere between 24 and 35%. Furthermore, saturation as a measure of estimated oximetry might have difference by as much 4%, therefore interpretation of SpO_2_ above 90% is very difficult due to the sigmoid shape of oxygen dissociation curve. For example, a saturation value of 95% results in a range of arterial oxygen tension (PaO_2_) anywhere between 60 and 200 mmHg. In this scenario, MR-proADM could add a useful piece of information for the correct triage of COVID-19 patients and also may become a useful tool in decision-making about enrolling patients into therapeutic clinical trials^23^.

Additionally, in accordance with previous studies, MR-ProADM assessment could accurately identify disease progression in patients with infection admitted to ED, safely increase out-patients management without increasing the number of re-admissions and/or mortality^9^, and identify patients requiring rapid admission to ICU^24^. In the same papers, the number of viral infections was very low, around 3%, but, considering the findings of our study, the hypothesis that MR-proADM might be a stable protein surrogate of severity in COVID-19 could be postulate as well. A level of MR-proADM < 0.89 nmol/l could be used together with clinical scoring in the ED rooms in COVID-19 patients as a safe admission avoidance tool with safety nets. However, further studies should be conducted to confirm this hypothesis.

This is the first study that have evaluated the serum level of MR-proADM in patients admitted to ED for SARS-COV-2 pneumonia and that correlates MR-proADM concentration to disease severity of COVID-19.

In fact, Haupf et al. have found an increase of adrenomedullin RNA expression in patients with more severe COVID-19 with respect to milder cases^12^. Further, ADM expression was not significantly different between patients with less severe COVID-19 and patients with other respiratory infections, postulating that endotheliitis might be one of the mechanisms involved in severe cases of COVID-19. In the same paper the ROC curve for ADM RNA was similar to the value found in our experience with measurement of MR-proADM protein^12^.

Montruccio et al. recently demonstrated that a higher mortality was found in patients with MR-proADM values higher than 1,8 nmol/L. In the logistic regression model, the odds ratio for mortality of MR-proADM was 10,2 and this biomarker had the best predictive ability with respect to age, gender, PCT, PCR, diabetes and cardiovascular diseases^13^.

In our cohort MR-proADM level was higher in patients with negative outcome, defined as the combination of death and IOT during hospitalization (1.38 [0.94–1.73] *vs.* 0.73 [0.56–0.94]). The multivariate analysis that evaluated factors influencing death or IOT shows that odds ratio of MR-proADM was 4.284 [1.893–11.413], together with neutrophil count (OR = 1.029 [1.011–1.049]) and WHO disease severity class (OR = 7.632 [5.871–19.496]).

In the work of Liu et al*.,* neutrophils were significantly higher in severe COVID-19 patients than in mild COVID-19 patients at the time of hospital admission. Their study showed that patients with severe COVID-19 had more serious lymphopenia and increased neutrophil count along with higher levels of circulating pro-inflammatory cytokines compared to patients with mild COVID-19^25^. The magnitude of increase in neutrophil count may suggest the intensity of inflammatory response in COVID-19 patients.

Many authors have postulated the cytokines storm as the mechanism involved in severe COVID-19 pneumonia and many therapeutic approaches have been targeted cytokines in order to reduce the inflammatory burden. Recently, some authors have also described that COVID-19 patients with interstitial pneumonia, but with no ARDS, have cytokine levels far lower than COVID-19 patients with ARDS. These differences were found with different methods of cytokines measurements and also using the same methods^4,5^. In our study, the MR-proADM value seems more accurate to identify patients with severe COVID-19 pneumonia with respect to cytokines, implying that endotheliitis might be more important than the cytokine storm at least in the early phase of the disease.

In this study, AUROC analysis showed a good discriminative performance of MR-proADM with respect to the combined event of death or IOT (AUROC: 0.849 [95% Cl 0.771–0.730], *p* < 0.0001). The optimal value of MR-proADM to discriminate negative outcome is 0.895 nmol/L with a sensitivity of 0.857 [95% Cl 0.728–0.987] and a specificity of 0.687 [95% Cl 0.587–0.787].

Limitations of this study are the retrospective nature of the data collection, the small number of patients included compared to the large number of COVID-19 patients that could have been evaluated, and the use of a single value of MR-proADM level instead of its kinetics during the whole hospitalization period.

## Conclusion

This study shows an association between MR-proADM levels and the severity of COVID-19. The assessment of MR-proADM could be of great value in triaging, evaluating possible escalation of therapies and admission avoidance or inclusion into trials.

Larger prospective and controlled studies are needed to confirm these findings, to understand if the level of MR-proADM might relate to critical care or not, and to investigate the diagnostic potential of MR-proADM as a marker of progression to severe COVID-19.

## Data Availability

The datasets used and/or analyzed during the current study are available from the corresponding author on reasonable request.

## Abbreviations

ACE2: Angiotensin converting enzyme 2
ADM: Adrenomedullin
ARDS: Acute respiratory distress syndrome
COVID-19: Coronavirus disease 2019
CRP: C-reactive protein
CK: Creatine kinase
ECs: Endothelial cells
FiO_2_: Fractional inspired oxygen concentration
ICU: Intensive care unit
IL: Interleuchine
IOT: Orotracheal intubation
LDH: Lactate dehydrogenase
LOQ: Limit of quantification
MR-proADM: Mid regional adrenomedullin
OR: Odds ratio
PaO_2_: Arterial oxygen tension
PCT: Procalcitonin
ROC: Receiver-operator characteristic
SARS-CoV-2: Severe acute respiratory syndrome coronavirus two
SIMEU: Italian society of emergency and urgency medicine
SOFA: Sequential organ failure assessment
